# P-1987. Development of Four qPCR Assays for the Detection and Differentiation of Dimorphic Fungal Pathogens

**DOI:** 10.1093/ofid/ofaf695.2154

**Published:** 2026-01-11

**Authors:** Clayton Thomas, James Grantham, Jamie Nutt, Steve Kleiboeker

**Affiliations:** Eurofins Viracor, Lenexa, Kansas; Eurofins Viracor, Lenexa, Kansas; Eurofins Viracor, Lenexa, Kansas; Eurofins Viracor, Lenexa, Kansas

## Abstract

**Background:**

Four pathogenic genera of fungi are found in multiple overlapping regions of the United States. These include *Blastomyces*, *Histoplasma, Coccidioides* and *Cryptococcus.* These pathogens are a significant cause of morbidity in immunocompetent and especially immunosuppressed patients. Differentiating members of *Histoplasma* and *Blastomyces* has proven difficult for Enzyme Immunoassays (EIAs). Identification based on polymerase chain reaction (PCR) can provide much-needed diagnosis with a quick turnover time.

**Methods:**

The primary objective of this study is to evaluate the ability of four in-house designed qPCR assays to detect, quantify and differentiate the specified fungal nucleic acid from each genus or species in BAL, sputum, and CSF specimens. Three assays were designed to detect, quantify and differentiate *Histoplasma*, *Blastomyces*, and *Coccidioides.* The *Cryptococcus* assay was designed to detect, quantify and differentiate between *C. gattii and C. neoformans.* All assays are quantitative and yield results in PCR copies/mL (c/mL). DNA was extracted with the MagMAX™ Prime Viral/Pathogen NA Isolation Kit and amplified by the Applied Biosystems™ Quant Studio 7 instrument.

**Results:**

The assays were validated in accordance with guidelines recommended by the New York State Department of Health, College of American Pathologists (CAP), and Clinical and Laboratory Standards Institute (CLSI) to establish analytical performance. All validation performance characteristics passed acceptance criteria including intra- and inter-assay precision and linearity and dynamic range. Limit of detection and lower limit of quantification was established for each assay. Analytical accuracy for all assays was within 0.5 log_10_ c/mL. 35 non-target fungal, bacterial, and viral pathogens were used to assess specificity and were negative for all fungal qPCR targets.

**Conclusion:**

Development of the Dimorphic Fungal qPCR Panel of assays aids in rapid diagnosis and provides insight into selection of appropriate antifungal therapies. The assays described here not only detect infections faster than other methods but provide accurate quantification allowing for rapid and targeted antifungal treatment options for the patient.

**Disclosures:**

Clayton Thomas, B.S., Eurofins Viracor: Employee James Grantham, B.S., Eurofins Viracor: Employee Jamie Nutt, n/a, Eurofins Viracor: Employee Steve Kleiboeker, PhD, Eurofins Viracor: Employee

